# Immune Evasion of Enteroviruses Under Innate Immune Monitoring

**DOI:** 10.3389/fmicb.2018.01866

**Published:** 2018-08-14

**Authors:** Ying Zhang, Jingyan Li, Qihan Li

**Affiliations:** Institute of Medical Biology, Yunnan Key Laboratory of Vaccine Research and Development on Severe Infectious Disease, Chinese Academy of Medical Sciences and Peking Union Medical College, Kunming, China

**Keywords:** innate immunity, enterovirus, immune evasion, *Picornaviridae*, innate lymphoid cells (ILCs)

## Abstract

As a major component of immunological defense against a great variety of pathogens, innate immunity is capable of activating the adaptive immune system. Viruses are a type of pathogen that proliferate parasitically in cells and have multiple strategies to escape from host immune pressure. Here, we review recent studies of the strategies and mechanisms by which enteroviruses evade innate immune monitoring.

## Introduction

Immunological defense mechanisms in vertebrates against various pathogens in the environment comprise innate immunity and adaptive immunity (Medzhitov and Janeway, 1997a,b; Hoebe et al., 2004). Innate immunity is capable of providing a persistent and non-specific physiological response to interrupt pathogenic invasion through a series of activated molecules and immune cells, including macrophages, neutrophils, natural killer (NK) cells, dendritic cells (DCs), and innate lymphoid cells (ILCs) (Medzhitov and Janeway, 1997a; Kleinnijenhuis et al., 2012). These cells are usually located in skin and mucosal tissues and are recruited to sites of pathogen infection by immunological signaling molecules secreted from infected or stimulated epithelia that recognize various pathogen-associated molecular patterns (PAMPs) through pattern-recognition receptors (PRRs) expressed on the cell membrane and cellular organelles (Modlin, 2012; Kawamura et al., 2014; Matejuk, 2018). This process initiates the activation of the immune system, which includes the expression of the first and second levels of immune-activating molecules, such as cytokines or chemokines, and the activation of T and B cells (Medzhitov and Janeway, 1997a; Kawamura et al., 2014). Innate immunity is the primary activator of the adaptive immune response.

Viruses, which are important vertebrate pathogens, show characteristic parasitic proliferation that depends on restrictive host cells and the development of systematic immune evasion mechanisms under host immune pressure over the long course of viral evolution (Finlay and McFadden, 2006; Ressing et al., 2015; Schulz and Mossman, 2016). These mechanisms include various virus-encoded molecules that are capable of targeting different immune receptors and signaling molecules and targeting functional innate and/or adaptive immune molecules to block the immune response; these interactions between viral and host components might lead to clinical and pathological outcomes in the body (Ploegh, 1998; Medzhitov, 2001). As a typical example of immune evasion, human immunodeficiency virus (HIV) uses five mechanisms to escape immune monitoring, including antigen evasion due to high variation in its genome (Borrow et al., 1997), evasion through integration of viral genes into the host genome (Lusic and Siliciano, 2017), inaccurate recognition of cytotoxic T lymphocytes (CTLs) on viral antigenic epitopes due to conformational changes in antigenic structure (Evans et al., 1999; Leviyang and Ganusov, 2015), structural mutability of major histocompatibility complex (MHC)-antigen epitope complexes by antigenic variation (Collins and Baltimore, 1999; Gorin et al., 2017), and lethal damage to CD4^+^ T cells caused by toxic viral proteins (Piguet and Trono, 2001; Sugden et al., 2017). These mechanisms are based on the tendency of RNA viruses to accumulate mutations in their genomes.

Enterovirus (EV) is a group of the *Picornavirus* family of non-enveloped, positive-stranded RNA viruses that contains more than 100 members, including poliovirus, coxsackievirus, echovirus, and many common enteric viral pathogens (Jubelt and Lipton, 2014). Humans are a natural host of enteroviruses (Solomon et al., 2010) and are usually infected through the fecal-oral and aerosol-respiratory routes, followed by viral shedding, as observed in throat and/or fecal swab assays several days after infection (Verboon-Maciolek et al., 2003; Han et al., 2010; Li et al., 2013). Clinical symptoms include fever, weakness, lethargy and diarrhea, and severe infections are associated with complications such as lesions and organ failure, including of the central nervous system, heart and liver, all of which may be observed in children (Lake et al., 1976; Wang et al., 1999; Verboon-Maciolek et al., 2008; Ooi et al., 2010). The high morbidity of enterovirus infections is notable, even though it is lower than that of fulminating infections. Severe infection with an enterovirus such as poliovirus was once a nightmare in the history of childhood infectious diseases due to irreversible paralysis, and the results of comprehensive studies of inactivated and attenuated poliovirus vaccines were a highly successful historic achievement (Chumakov et al., 2007; Reynolds, 2007). Although it is reasonable for humankind to be proud of this work, the occurrence of vaccine-associated paralysis poliomyelitis (VAPP) and vaccine-derived poliovirus (VDPV) suggests that immune evasion is an important biological characteristic of enteroviruses (Kew et al., 2004; Kew et al., 2005; Minor, 2009). In addition, epidemiological analyses have provided data showing constant pandemic infection with enteroviruses, such as human foot-hand-mouth disease and viral cardiomyopathy (Blomberg et al., 1974; Wenner, 1982; Ooi et al., 2010). In this case, prophylactic or curative treatments of diseases induced by enteroviruses should be developed based on an understanding of viral immune evasion strategies during infection.

To further understand the immunological escape characteristics of enteroviruses, especially their interactions with innate immunity, a brief review is presented here based on our knowledge and experience in enterovirus studies.

## Enterovirus Evasion of Identification by Host Innate Immunity

An immunological study indicated that host cells usually identify external pathogens through their PRRs, which recognize PAMPs (Medzhitov and Janeway, 1997a; Saito and Gale, 2007; Stuart et al., 2013). Five PRRs, include toll-like receptors (TLRs) (Athman and Philpott, 2004; Saito and Gale, 2007; Kawai and Akira, 2010), RIG-I-like receptors (RIG-I) (Thompson and Locarnini, 2007; Fredericksen et al., 2008; Wilkins and Gale, 2010), NOD-like receptors (NLRs) (Kanneganti, 2010; Wilkins and Gale, 2010; Kufer and Sansonetti, 2011), AIM2-like receptors (ALRs) (Yang et al., 2015), and the cGAS/STING system (Cai et al., 2014; Ma and Damania, 2016), have been shown to recognize viral PAMPs. TLRs, which include 10 members in humans and 13 in mice, were the first group of PRRs identified and are responsible for sensing invasive pathogenic stimuli signals (Meylan et al., 2006; Sansonetti, 2006). TLRs are evolutionally conserved proteins that are distributed extracellularly or intracellularly in various cells, especially immune cells and epithelial cells, highlighting their importance in the process of host antiviral immunity (Anders and Schlondorff, 2007). TLR-3, -4, -7, -8, -9 are involved in antiviral immunity, and after binding by viral fragments of DNA or RNA and proteins, these TLRs are capable of initiating conformational change in the structure of extracellular and cytoplasmic domains to recruit adaptor molecules in their signaling pathways, which sequentially activate the core component NF-κB and AP-1 motif in the innate immune response (O’Neill et al., 2013; Kawamura et al., 2014). As part of the same mechanism, the other PRRs, including RIG-I and NLRs, are also responsible for sensing intracellular viral pathogenic signals and transferring the stimulus to the NF-κB motif (Jeong and Lee, 2011; Oviedo-Boyso et al., 2014). This comprehensive process has been identified as a key step for innate immunity and cooperation (Meylan et al., 2006; Hansen et al., 2011). This cooperation is modulated systematically and closely with pathogenic stimulation and the release of signaling molecules for the formation of adaptive immunity.

### Enterovirus Evasion of Extracellular Recognition

**FIGURE 1:**
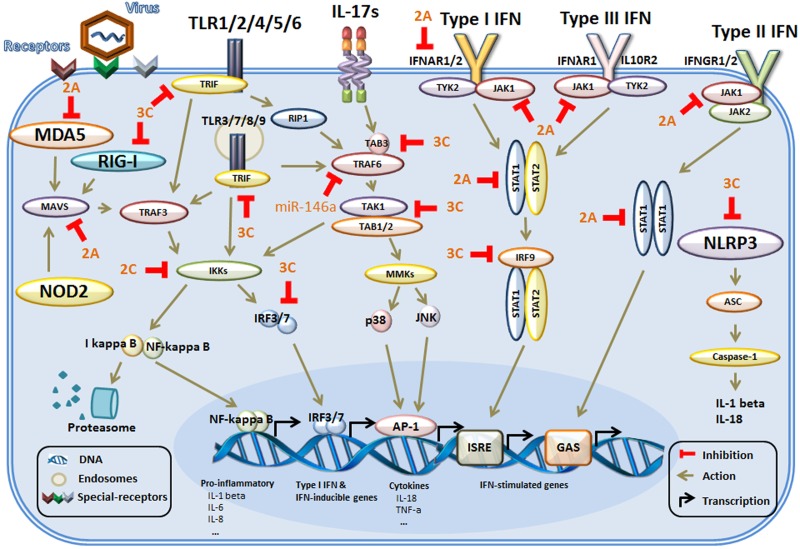
The main mechanism of innate immunity evasion by enteroviruses in host cells. Host cells identify external pathogens through their PRRs, including TLRs, RIG-I, and NLRs. The PRRs initiate conformational changes and recruit adaptor molecules in their signaling pathways. Then, the downstream signals are propagated through the activation of core components, such as TRAF6 and the IKK complex, culminating in the activation of transcription factors, which regulate the production of genes associated with the innate immune response. In addition, during binding of IFNs to the IFN receptors, multiple signaling pathways are activated. Then, activation of the kinases and downstream formation of various STAT complexes mediates IFN-dependent gene transcription of IFN-stimulated genes. The adapter proteins and key kinases that mediate these signaling pathways are targeted by viral proteins (such as 2A, 2C, and 3C) or microRNAs (miR-146a). TLRs, toll-like receptors; RIG-I, RIG-I-like receptors; NLRs, NOD-like receptors; NOD2, nucleotide binding oligomerization domain 2; MDA5, melanoma differentiation gene 5; MAVS, mitochondrial antiviral signaling protein; NLRP3, NOD-like receptor 3; TIRAP, toll-interleukin 1 receptor (TIR) domain-containing adaptor protein; TRIF, TIRAP inducing IFN-β; IB, inhibitor of NF-κB; IKKs, IB kinases; STAT, signal transducer and activator of transcription factor; TRAF6, tumor-necrosis-factor- receptor-associated factor 6; JNK, c-Jun N-terminal kinase; IRFs, interferon-regulatory factors; ASC, apoptosis-associated speck-like protein containing a CARD (caspase recruitment domain); ISRE, IFN-stimulated response element); GAS, IFN-γ activation sequence.

After breaking through the physical barrier of the skin, the first priority of enteroviruses is to avoid recognition by the innate immune system. Evasion is very difficult for viruses because of the wide distribution of TLRs on the surfaces of more than 20 types of immune cells, especially innate immune cells, including monocytes, NK cells, macrophages, ILCs, DCs (Fearon and Locksley, 1996; Geissmann et al., 2010; Galli et al., 2011), and adaptive immune cells, including T and B lymphocytes and even epithelial cells (Medzhitov and Janeway, 1997a; Schleimer et al., 2007; Hammad and Lambrecht, 2008). Five members of the TLR family are involved in the recognition of viral PAMPs (Stuart et al., 2013; Yang et al., 2015; Ma and Damania, 2016). To escape recognition by TLRs and find shelter for replication, enteroviruses use various means, which are described below (**Figure 1**).

#### Binding to Specific Receptors

Usually, various physiological receptors are expressed in cells for transduction of specific signals and other functions (Aplin et al., 1998; Medzhitov, 2001; Lefkowitz and Shenoy, 2005). Viruses strategically use these receptor molecules that are expressed physiologically in the cell membrane to mediate their entry (Doranz et al., 1996; Agnello et al., 1999; Parker et al., 2001). Many cellular receptors, especially those expressed in immune cells to regulate immune function, are enterovirus-specific receptors (Canton et al., 2013). Previously, CD155 (also called nectin-like molecule, among other names) was found to interact specifically with poliovirus as its receptor (Belnap et al., 2000b; He et al., 2000) and function in the cell-mediated immune response via binding to CD226 (Lozano et al., 2012, 2013). Scavenger receptor class B member 2 (SCARB2) interacts with enterovirus type 71 (EV71), which mediates its entry into epithelial cells and some immune cells expressing this protein (Yamayoshi et al., 2009). The human T cell immunoglobulin and mucin domain 1 (Tim1) protein were identified as specific receptors for hepatitis A virus (HAV) (McIntire et al., 2003; Kim et al., 2011). Accumulative studies suggest that many proteins that are expressed on the surfaces of epithelial and immune cells that play different cellular biological roles, including adhesion, migration, and immune regulation, are also capable of binding to members of the enterovirus family (Rossmann et al., 2002). P-selectin glycoprotein ligand-1 (PSGL-1) is capable of enhancing EV71 and Coxsackievirus A type 16 (CA16) entry into cells after interaction with SCARB2 and PSGL-1 (Nishimura et al., 2010; Nishimura and Shimizu, 2012). Neuron-specific intercellular adhesion molecule 5 (ICAM-5/telencephalin) assists enterovirus 68 (EV68) binding and entry into cells (Wei et al., 2016). Additionally, SA-a-2,3-Gal and SA-linked O-glycan of sialylated glycans can act as receptors to co-mediate infection by Coxsackievirus A type 24 (CA24) and EV71 (Yang et al., 2009; Mistry et al., 2011).

Due to the distributions of these molecules on the surfaces of various cells, the binding of enteroviruses to these molecules not only mediates viral entry into cells but also accelerates viral evasion of recognition by the innate immune system.

#### Using the Internalization Process of Immune Cells to Escape Immune Recognition

As a basic immune defense function, immune cells are capable of initiating internalization processes to clear pathogens by recognizing pathogen PAMPs and damage-associated molecular patterns (DAMPs) to aggregate phagosomes (Mellman et al., 1986; Michallet et al., 2013). However, this immunological strategy is also utilized by viruses for entry into cells; for example, poliovirus and HAV change the conformation of the 160S capsid, leading to the formation of a cell-entry intermediate (Bergmann et al., 1997; Belnap et al., 2000a) known as the 135S or A-particle that subtly changes the pH environment in the cellular phagosome (Curry et al., 1996; Huang et al., 2000). This process helps the virus release its genome into the cytosol for replication. CA9 employs a similar strategy by using the cellular protein GRP78, a member of the heat shock protein 70 (HSP70) family, to assist MHC I in mediating virus internalization (Triantafilou et al., 2002). Echo 1 is capable of utilizing cellular multivesicular bodies (MVBs) as carriers for internalization into cells (Karjalainen et al., 2011). All of these data suggest that this is a strategy used by members of the enterovirus family to evade recognition by the innate immune system.

### Enterovirus Evasion of Intracellular Recognition by the Innate Immune System

Although most members of the enterovirus family cause cytopathic infection in various cells, they still require new tactics to elude innate immune monitoring *in vitro* after avoiding extracellular monitoring (**Figure 1**).

#### Interference With the TLR Pathway

Immunological studies have shown that when a TLR successfully recognizes a pathogenic molecule, it initiates a conformational change signal and gradually activates each molecule in the signaling pathway for signal transfer (West et al., 2006). Furthermore, the signals transferred from this pathway are able to activate the core component of innate immunity, namely, the NF-κB transcriptional complex, which enables the expression of various immune functional factors, including IFN-α, -β, -γ and other cytokines or chemokines (Muzio et al., 2000; West et al., 2006). These factors and the immune cells they activate have systematic antiviral effects *in vitro* and enable the restriction of viral proliferation (West et al., 2006). Enteroviruses encode specific proteins that interact with key molecules in this signaling pathway and interfere with signal transduction for the immune response. The non-structural EV71 component protein 3C is a typical example; it is capable of hydrolyzing specific peptide bonds in viral or cellular proteins, not only at the main site, Cys/Ser (or Gly), but also at other cleavage sites in a few cases (Laitinen et al., 2016), potentially leading to the inactivation of several proteins in the TLR pathway, including TRIF, TAK1, and IRF7 (Cui et al., 2011; Lei et al., 2011, 2013, 2014). Obviously, the result of this process is inhibition of NF-κB and AP-1 transcriptional systems modulated by MMKs or IRF3/7, leading to inhibited expression of sets of genes, such as those of inflammatory factors, cytokines, and the interferon (IFN) family, which is related to innate immunity (Baeuerle and Baichwal, 1997; Zeytun et al., 2007). Another EV71 protein, 2C, is also involved in viral interference with the TLR pathway by blocking IKKα/β, which impacts the NF-κB and IRF3/7 transcriptional systems (Zheng et al., 2011; Li et al., 2016). Additionally, EV71 enables RNA interference with the TLR pathway, in which an miRNA is capable of inhibiting TRAF6 and reducing the efficiency of the innate response *in vitro* (Ho et al., 2014; Hu et al., 2016). HAV, poliovirus, and CB3 are all capable of targeting similar proteins in the TLR pathway using similar mechanisms (Mukherjee et al., 2011; Qu et al., 2011).

#### Interference With the RIG-I Pathway

As the major adaptor in the RIG-I pathway of mammalian cells, RIG-I is a key component of virus recognition in this pathway and interacts with viral RNA containing the 5′-ppp terminal structure (Takeuchi and Akira, 2008; Schmidt et al., 2009; Wang et al., 2010). This feature allows RIG-I to effectively recognize members of the *Orthomyxoviridae, Paramyxoviridae*, and *Flaviviridae* families (Yoneyama et al., 2004; Kato et al., 2006; Yoneyama and Fujita, 2009). Another key adaptor, MDA5, is thought to recognize long viral double-stranded RNA molecules (Takeuchi and Akira, 2008), although its mechanism is unclear. MDA5 sensitively detects members of the *Picornaviridae* and *Coronaviridae* families (Yoneyama and Fujita, 2009). To evade immune monitoring by the RIG-I pathway, enteroviruses encode a small protein, Vpg, that binds to the 5’-ppp end of its RNA genome and allows the RNA to escape molecular recognition by RIG-I (Yoneyama and Fujita, 2009). In addition, viruses induce the formation of stress granule (SG)-like aggregates, termed antiviral SG (avSG), in the cytoplasm to escape recognition by MDA5 (Yoneyama et al., 2015). Furthermore, enteroviruses might utilize various encoded proteins to block or interrupt the RIG-I pathway, such as the 3C protein, which is encoded by poliovirus and EV71 and is capable of cleaving RIG-I (Barral et al., 2009; Feng et al., 2014). The cleavage of MDA5 is enabled via EV71-encoded non-structural protein 2A or the proteasome and caspases induced by poliovirus (Barral et al., 2007; Feng et al., 2014). During EV71, coxsackieviruses and polio infection, host MAVS/IPS-1 is cleaved by viral proteins (Mukherjee et al., 2011; Feng et al., 2014; Kotla and Gustin, 2015; Lind et al., 2016).

#### Interference With the NLR Pathway

The NLR family can be divided into the NOD, NLRP, and IPAF subfamilies based on the type of central NACHT domain, which is responsible for activation and oligomerization (Schroder and Tschopp, 2010).

##### Interference with the NOD2 pathway

NOD2 recognizes peptidoglycan motifs from bacterial cells and intracellular muramyl dipeptides (Girardin et al., 2003). NOD2 signaling is disrupted when its downstream signaling partially overlaps with that of the RIG-I pathway (Ramos and Gale, 2011). The immune gene transcription system mediated by NF-κb and IRF3/7 is perturbed, leading to changes in the innate immune response after the EV71-encoded 2A protein inhibits MAVS/IPS-1, the 2C protein inhibits IKKs, and the 3C protein inhibits IRF3/7 (Lei et al., 2013; Feng et al., 2014; Li et al., 2016).

##### Interference with the NLRP3 pathway

The NLRP3 inflammasome, which is a critical component of innate immunity that senses various pathogens and DAMPs (Schroder and Tschopp, 2010), is capable of controlling the maturation and secretion of the proinflammatory cytokines IL-1β and IL-18 (Schmidt and Lenz, 2012). NLRP3 induces an effective immune response to defend the host from EV71 and polio infection, whereas the NLRP3 inflammasome is regulated via viral proteins produced by enteroviruses (Ito et al., 2012; Wang et al., 2015). An *in vitro* cellular study indicated that inflammasome activation is suppressed by the cleavage of NLRP3 by EV71-encoded 2A and 3C proteases (Wang et al., 2015; Yu et al., 2017). Additionally, this study demonstrated that the EV71 3C protease interacts with NLRP3 and suppresses the secretion of IL-1β in mammals, which affects the innate immune response (Wang et al., 2015). Non-structural protein 2B of poliovirus and EV71 can induce redistribution of NLRP3, thus affecting activation of innate immunity (Ito et al., 2012).

#### Interference With the IL-17 Pathway

As a proinflammatory cytokine, IL-17 is produced by innate immune T helper cells (Th 17 cells) and is capable of inducing downstream transcription and expression of immune genes based on activation of the NF-κb and MAPK pathways (Hata et al., 2002; Korn et al., 2009). Thus, EV71 infection regulates core processing of the IL-17 pathway because it overlaps with TLRs and the RIG-I pathway, as described above.

## Enterovirus Evasion of Antigen Presentation by Host Mhc

Major histocompatibility complex, a cell surface protein, is essential for recognizing and triggering the T cell immune response by binding to peptides cleaved from pathogens (Hughes and Nei, 1988). The MHC family is divided into three subgroups (MHC class I, II, and III) based on structure, function and distribution. Class I MHC (MHC I) occurs on all nucleated cells, and class II occurs on only professional antigen-presenting cells (APCs) and is directly related to virus infection and antigen presentation (Hughes and Nei, 1988; Inaba et al., 1993). By presenting virus-specific peptide segments on the cell surface, MHC initiates the activation of CTLs to kill infected cells, prevent virus replication and release, and thus assist in the elimination of virus from the host (Schwartz, 1985).

Enteroviruses have different strategies for suppressing the presentation of peptides by MHC at different stages for evasion from CTL recognition as follows.

A typical example is Coxsackievirus B3, which contains at least three different viral proteins, including viral protein 3A, which interrupts the transportation of complexes to the Golgi apparatus (Wessels et al., 2005), as well as 2B and 2BC, which activate autophagy to rapidly remove proteins from the cell surface to inhibit the presentation of MHC I on the cell surface for host immune evasion (Cornell et al., 2007). This virus not only interferes with the presentation of MHC I on the cell surface but also induces the production of an efficient inhibitor of IFNγ-induced MHC II expression-IK, leading to the inhibition of MHC II presentation (Park et al., 2013). Similarly, poliovirus utilizes the 3A protein to suppress MHC I-dependent antigen presentation (Deitz et al., 2000). Additionally, MHC II deficiency is the most common diagnosis (54%) in enterovirus-positive patients (Driss et al., 2012). Although the reason and mechanism by which the EV71 virus renders MHC II ineffective during infection is not yet clear, all evidence suggests that enteroviruses are capable of evading the innate immune response in specific ways.

## Inhibition of the Ifn Response

Interferons are a group of active proteins that function as broad-spectrum antivirals (Tomoda et al., 2001; Liu et al., 2012). Instead of directly killing or inhibiting viruses, IFNs activate receptors on the cell surface to produce antiviral proteins to eradicate viruses (Fensterl and Sen, 2009). In addition, IFNs are capable of activating immune cells, including NK cells, macrophages and T lymphocytes, and increasing host defenses by regulating the immune system (Djeu et al., 1979; Murray, 1988; Le Page et al., 2000). IFNs can be divided into three classes, type I IFN, type II IFN, and type III IFN, based on amino acid structure, antigenicity, and secretory cells (Chelbi-Alix and Wietzerbin, 2007).

### Induction of IFNs

In response to virus-specific antigen molecules (DNA or RNA), innate immune cell receptors are able to activate the transcription factor IRF3/7 and induce type I IFN through signaling cascades (Sato et al., 2000). Enteroviruses can impede core components of these pathways with non-structural proteins (e.g., 2A, 2C, 3C) or microRNAs (e.g., miR-146a) to prevent type I IFN production (Lei et al., 2010; Ho et al., 2014).

Type II (γ) IFN is released by APCs (Frucht et al., 2001). There is no obvious evidence that enterovirus directly inhibits IFN-γ production.

### Interference With IFN Pathways

The antiviral function of the IFN pathway is realized by binding to specific cell surface receptors. Receptors that can recognize IFNs are divided into three types: type I IFN receptors consist of IFNAR1 and IFNAR2 (de Weerd et al., 2007), type II IFN receptors consist of IFNGR1 and IFNGR2 (Schroder et al., 2004), and type III IFN receptors consist of IFNλR1 and IL10R2 (Zhou et al., 2011). Signaling cascades are activated downstream to regulate the innate immune response after IFN receptors interact with specific IFN signaling molecules. Enteroviruses have evolved mechanisms to escape this complex but effective innate immune response at different stages by interacting with different core molecules. EV71, for example, is capable of regulating signaling pathways at the initial cell surface signaling stage by cleaving eIF4G to prevent IFNAR1 production of the type I IFN receptor via viral protein 2A to reduce the expression of the type I IFN receptor and disrupt the type I IFN pathway (Lu et al., 2012). Moreover, viral protein 2A blocks phosphorylation of JAK1, TYK2, STAT1, and STAT2, members of the JAK-STAT pathway, resulting in attenuation of immune signaling (Liu et al., 2014). Furthermore, the cleavage of the IRF9 protein by viral protein 3C further inhibits JAK-STAT signaling and eventually affects the transcription of IFN-induced genes to resist activation of the innate immune response (Hung et al., 2011).

## Enteroviruses Interact With Ilcs

Innate lymphoid cells are a recently discovered subset of lymphocytes that lack antigen-specific B or T cell receptors because of the absence of the recombination activating gene (Rag) (Moretta and Locatelli, 2016). ILCs are widely found in tissues and organs, particularly in mucosal tissues and lymphoid organs (Spits et al., 2013). ILCs are usually activated within hours after infection and play an important role in modulating innate and adaptive immune responses (Gasteiger and Rudensky, 2014). Based on their function in immunity against pathogens, ILCs respond to the initial invasion of pathogens and mediate innate immune signaling to activate adaptive immunity (Spits et al., 2013). Currently, much concern about ILCs focuses on understanding the role of ILCs in assisting the induction of specific immune responses.

Our research investigating the role of ILCs in enterovirus infection, including CA16, EV71, and CA10, has provided interesting data. These viruses show similar pathogenic characteristics of infection of the respiratory or gut epithelium followed by typical viremia and viral proliferation in various organs, leading to clinical pathogenic processes characterized by blisters on the hands, feet and mouth associated with other clinical manifestations, such as fever and flu-like symptoms (Liu et al., 2011; Wang et al., 2017). However, the immunity induced during infection with these viruses presents obvious differences in efficacy, especially their clinical protective efficacy against viral attack (Li et al., 2011; Wang et al., 2017). Our recent unpublished work has suggested that CA16-infected epithelial cells play a role in initiating the transduction of antigenic stimulus signals from local tissue to ILCs and activation of these ILCs, which were found to be co-localized with viral antigen in tissues. Similarly, EV71 infection is co-localized with ILC2 in respiratory mucosal tissue, in contrast to the co-localization of CA16 infection with ILC1 and 3. Although our data do not clearly explain the relationships between viral infection and these differences in localization and the induced immune response, the elucidation of the significance of the interactions between enteroviruses and ILCs might provide novel insights.

## Conclusion

In summary, the data obtained to date suggest that interactions between enteroviruses and the immune system are complicated and that enteroviruses employ various ingenious tactics by targeting different molecules in different pathways. To understand the systematic process of virus infection, more work is still needed.

## Author Contributions

YZ and QL wrote this review. JL modified the manuscript. YZ wrote the first draft.

## Conflict of Interest Statement

The authors declare that the research was conducted in the absence of any commercial or financial relationships that could be construed as a potential conflict of interest.
